# Supplementary material for: Identification of photoperiod-induced specific miRNAs in the adrenal glands of Sunite sheep (Ovis aries)
Source: Front Vet Sci. 2022 Jul 22;9:888207. doi: 10.3389/fvets.2022.888207 (PMC9354845; doi:10.3389/fvets.2022.888207)
Supplement: Supplementary file 1 [file Data_Sheet_1.ZIP › Supplementary file/miRDeep2 file.zip!/miRDeep2 file/Novel_Structure/SP42A1/Novel_470.pdf]

| | | |
|---|---|---|
| Provisional ID | : | NC_019484.2_26986 |
| Score total | : | 47.1 |
| Score for star read(s) | : | 3.9 |
| Score for read counts | : | 39.9 |
| Score for mfe | : | 1.7 |
| Score for randfold | : | 1.6 |
| Score for cons. seed | : | |
| Total read count | : | 90 |
| Mature read count | : | 85 |
| Loop read count | : | 0 |
| Star read count | : | 5 |

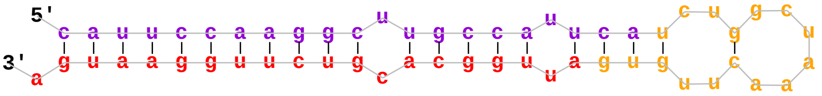


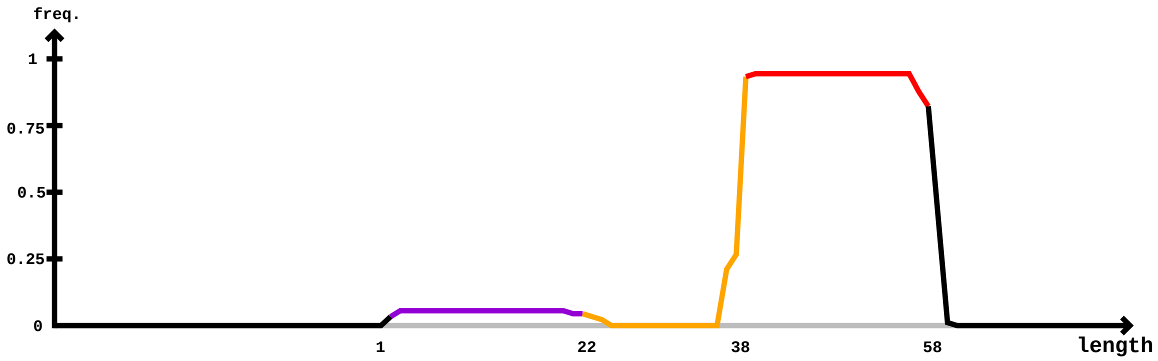


```
                                  Star                                 Mature
5'- uugccaccuucagcgugucugugugcuguaccccauuccaaggcuugccauucaucuggcuaaacuugugauuggcacgucuuggaaugaaguaacguacaacaaugaug  -3'   obs
    uugccaccuucagcgugucugugugcuguaccccauuccaaggcuugccauucaucuggcuaaacuugugauuggcacgucuuggaaugaaguaacguacaacaaugaug        exp
    ..((........))..((((((((((..(((..(((((((((((.(((((.((((..(......)..)))).))))).)))))))))))..)))..)))).)))..))).  reads   mm   sample
    .................................cauuccaaggcuugccauu..........................................................  1       0    seq
    .................................cauuccaaggcuugccauuca........................................................  2       0    seq
    ..................................auuccaaggcuugccauucauc......................................................  2       0    seq
    ....................................................................ugauuggcacgucuuggaau......................  4       0    seq
    ....................................................................ugauuggcacgucuuggaaug.....................  3       0    seq
    ....................................................................ugauuggcacgucuuggaauga....................  11      0    seq
    ....................................................................ugauuggcacgucuuggaaugaa...................  1       0    seq
    .....................................................................gauuggcacgucuuggaauga....................  5       0    seq
    ......................................................................auuggcacgucuuggaau......................  2       0    seq
    ......................................................................auuggcacgucuuggaaug.....................  2       0    seq
    ......................................................................auuggcacgucuuggaauga....................  41      0    seq
    ......................................................................auuggcacgucuuggaaugaa...................  14      0    seq
    ......................................................................auuggcacgucuuggaaugaag..................  1       0    seq
    .......................................................................uuggcacgucuuggaauga....................  1       0    seq
```
